# Chemical Composition of Selected Commercial Herbal Remedies in Relation to Geographical Origin and Inter-Species Diversity

**DOI:** 10.1007/s12011-017-1078-z

**Published:** 2017-06-21

**Authors:** Pawel Konieczynski, Agnieszka Viapiana, Roman Lysiuk, Marek Wesolowski

**Affiliations:** 1 Department of Analytical Chemistry, Medical University of Gdansk, Gen. J. Hallera 107, 80-416, Gdansk, Poland; 2 Department of Pharmacognosy and Botany, Danylo Halytsky Lviv National Medical University, Pekarska 69, Lviv, 79010 Ukraine

**Keywords:** Essential elements, Total flavonoids, Herbal remedies, Statistical evaluation

## Abstract

Infusions prepared from medicinal herbs that are rich in flavonoids are very popular herbal remedies in societies of Eastern Europe. Therefore, the content of essential elements together with total flavonoids was analyzed in 65 commercially available samples of herbal drugs originating from Ukraine, Romania, and Belarus. The results showed that metallic elements (in mg kg^−1^ d.w.) have occurred in the following order: Fe > Mn > Zn > Cu, both for total and water-extractable species. Total flavonoids were determined in the range from 10.0 to 191.8 mg g^−1^ d.w. Several significant correlations have been found between the analytes, especially among water-extractable Fe with other metals, and total flavonoids and Fe, Zn, and Mn. Analysis of variance has revealed significant differences among studied samples due to their origin from different countries, especially between Belarussian samples and others. Differences owing to belonging to various plant species were also found, as it was noticed in the case of *Polygoni aviculare herba* in comparison with other botanical plant species. Moreover, multivariate statistical techniques, such as cluster analysis (CA) and principal component analysis (PCA) were used to gather herbal drugs based on similarity of chemical composition. CA grouped the samples into clusters with similar level of elements and total flavonoid contents, and PCA has indicated *Hyperici herba*, *Tiliae flores*, and *Crataegi fructus* as herbal remedies with close concentration of studied elements and flavonoids.

## Introduction

Drugs of natural origin, including preparations obtained from medicinal herbs, are still in use in many countries of the world. They are considered as safer in comparison with synthetic drugs, and they have less adverse effects for human organism. Among herbs used in medicine, there are remedies containing rich concentration of flavonoids, known as antioxidant compounds, which have numerous beneficial effects on human organism [1–5]. Not counting flavonoids, which are secondary metabolites in plant kingdom, medicinal plants can be ubiquitous sources of essential elements, too [6–11]. These elements, especially metals, play indispensable role in metabolic processes of living organisms by participation in important biochemical transitions as co-factors of enzymes [12].

In Eastern Europe, the use of medicinal herbs is very popular. In Ukraine, Lithuania, Poland, Belarus, Serbia, Bulgaria, and Romania, people often collect medicinal plants from nature and prepare infusions, decoctions, or herbal teas for themselves to treat various diseases, such as common cold, anxiety, and nausea. Drugs of natural origin have less adverse effects, and their beneficial action on human organism is caused by whole complex of secondary metabolites, as well as it is enhanced by numerous macro- and microelements.

There were many studies, in which level of flavonoids, as well as concentration of essential elements in herbal drugs were investigated [13–16]. Essential elements and their relations to phenolic compounds in infusions of medicinal plants from various European regions (Lithuania, Serbia, Italy, and Portugal) were thoroughly studied by spectroscopic and chromatographic techniques. It was revealed that medicinal plant infusions differed significantly, strongly depending on plant species, regardless of the origin from distant geographical areas [11]. However, by using ICP-OES (inductively coupled plasma–optical emission spectrometry) and LIBS (laser-induced breakdown spectroscopy) techniques to study elemental composition in herbs rich in flavonoids form Poland, Lithuania, and Serbia, it was found that there is no significant impact of geographical origin on elements levels [13].

On the other hand, element concentrations of different species of *Salvia officinalis* were studied and it was determined that the elemental contents were statistically different between the species and within one species, depending strongly on the place of collections [14]. Total phenolic and flavonoid concentration together with several elements were investigated in seeds and aerial parts of *Trigonella monspeliaca*, a medicinal plant commonly cultivated in Mediterranean region. This study has shown that the seed extract contained significantly higher amount of total phenolics, whereas aerial part of the studied plant had high level of total flavonoids [15]. Other researchers investigated antioxidant activity and also elemental, phenolic, and flavonoid contents in lemon grass, and they revealed that potassium was the most abundant element, whereas copper was the least [16]. Moreover, concentrations of eight studied elements differed significantly from one location to another [16].

All of these have led us to the aim of studies, which was to determine the chemical composition of herbs originating from Ukraine, Belarus, and Romania in order to detect similarities and differences in the contents of total flavonoids and of selected essential elements. Therefore, concentrations of Fe, Mn, Zn, and Cu were determined both in the digests obtained from nine different botanical plant samples, and in their aqueous infusions. To fulfill the aim of this study, multivariate statistical methods were used.

## Materials and Methods

The studied medicinal herbs listed in Table 1 were purchased at herbal shops in Ukraine, Romania, and Belarus. They represent nine botanical plant species rich in flavonoid contents. Prior to analysis, the plant samples were ground in a Knifetec sample mill 1095 (Höganäs, Sweden) and kept in polyethylene containers up to the analyses.

**Table 1:** The list of studied medicinal herbs from Ukraine, Romania, and Belarus

No.	Herbal remedy	Serial number	Country of origin
1	*Crataegi folium et flores*	0010115	Ukraine
2		20615	Ukraine
3		31505663	Romania
4		150903	Romania
5		E 27082017	Romania
6		110116	Belarus
7	*Crataegi fructus*	050615	Ukraine
8		0010314	Ukraine
9	*Helichrysi arenarii flores*	020315	Ukraine
10		100715	Ukraine
11		530315	Ukraine
12		10515	Ukraine
13		08201015	Belarus
14	*Sambuci nigrae flores*	0010315	Ukraine
15		20315	Ukraine
16		31505528	Romania
17		150904	Romania
18		E 11082017	Romania
19		820715	Belarus
20	*Chamomillae flos*	120715	Ukraine
21		0150515	Ukraine
22		110615	Ukraine
23		060215	Ukraine
24		50715	Ukraine
25		31505757	Romania
26		153303	Romania
27		E16092017	Romania
28		06090216	Belarus
29		1331115	Belarus
30	*Tiliae flores*	0010215	Ukraine
31		20315	Ukraine
32		010215	Ukraine
33		050315	Ukraine
34		31505858	Romania
35		153303	Romania
36		E17092017	Romania
37	*Violae herba*	0020414	Ukraine
38		20415	Ukraine
39		020315	Ukraine
40		E30072017	Romania
41		280315	Belarus
42	*Hyperici herba*	050515	Ukraine
43		050515	Ukraine
44		050515	Ukraine
45		0010315	Ukraine
46		130815	Ukraine
47		20215	Ukraine
48		31505730	Romania
49		153101	Romania
50		E28092017	Romania
51		73011215	Belarus
52	*Polygoni aviculare herba*	030315	Ukraine
53		030315	Ukraine
54		21113	Ukraine
55		31403803	Romania
56		E14042017	Romania
57		010715	Belarus
58	*Equiseti herba*	0010315	Ukraine
59		20615	Ukraine
60		060615	Ukraine
61		020315	Ukraine
62		020315	Ukraine
63		31505656	Romania
64		153701	Romania
65		E25092017	Romania

To prepare the herbs for determination of total concentrations of the studied metallic elements, a sample (about 1.0 g) was digested with a mixture of HNO_3_ (65% Selectipur solution) and H_2_O_2_ (30% solution) (3 + 5, *v*/*v*) (Merck, Germany) using the microwave-assisted digestion procedure (UniClever BM-1z, Plazmatronika Wrocław, Poland).

For infusion making, a sample of a medicinal herb (about 2 g) was placed in a 250-mL beaker, and 100 mL of boiling deionized water obtained in double-distillation system (Heraeus Quarzglas, Germany) was added. Then, the sample was brewed for 15 min under glass cover, and solution was filtered through medium size filter paper. The obtained extract was transferred into a volumetric flask and diluted up to 100 mL with redistilled water.

Spectrophotometric UV/Vis method based on procedure described earlier [17] was used for total flavonoid determination applying SP-870 spectrophotometer (Metertek, South Korea). Flame-atomic absorption spectroscopy (A250 plus spectrometer, Varian, Australia) was applied to determine Fe, Mn, Zn, and Cu concentrations both in digests obtained from medicinal plants, and in aqueous extracts (infusions) prepared from the studied herbal materials.

Statistical calculations—correlation analysis and cluster (CA) and principal component (PCA) analyses—were performed using Statistica 10 software (StatSoft Inc., Tulsa, USA) on the basis of parametric tests with the level of significance of *p* < 0.05 [18]. Analysis of variance (ANOVA) test followed by Tukey post hoc test was performed to check significant differences between the studied samples.

## Results and Discussion

**Fig. 1:**
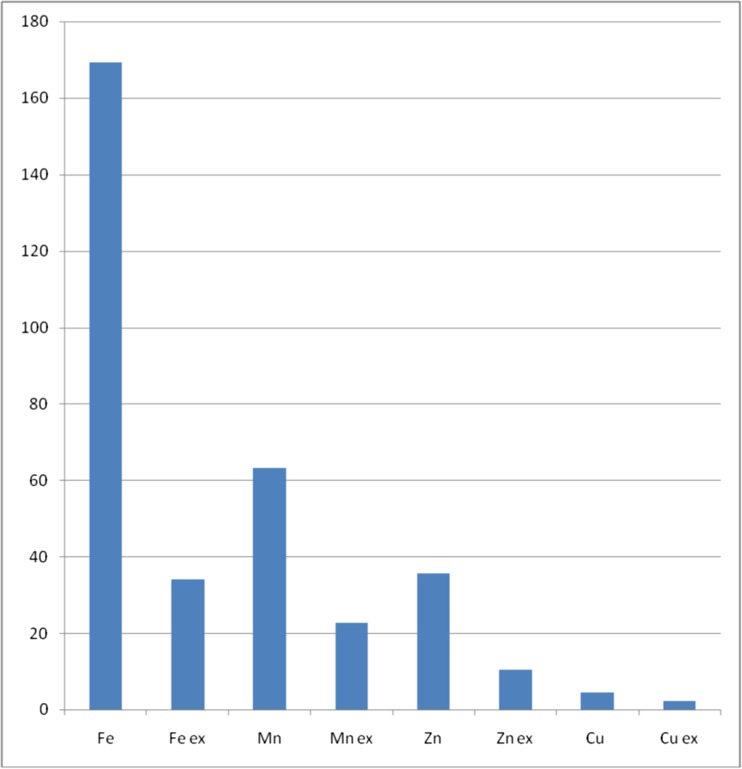
Mean (*n* = 65) concentration of metallic elements in all studied herbal drugs (mg kg^−1^ d.w.). Total and water-extractable forms (ex) are shown

Results of determination of four essential metals, both their total concentration and water-extractable forms, together with that of total flavonoid contents, are presented in Table 2. Levels of metallic elements were determined in all studied herbal drugs in the following order: Fe > Mn > Zn > Cu, both for total and water-extractable species. This tendency is clearly illustrated in Fig. 1.

**Table 2:** Results of determination of metallic elements (mg kg^−1^ d.w.) and total flavonoid content (mg g^−1^ d.w.) in studied medicinal herbs

No.	Herbal remedy	Total Fe	Fe ex	Total Zn	Zn ex	Total Mn	Mn ex	Total Cu	Cu ex	Total flavonoids
1	*Crataegi folium et flores*	101.31	8.30	31.81	8.41	23.50	2.75	3.05	1.65	17.98
2		90.43	6.58	44.43	11.83	21.06	10.31	2.41	1.83	112.16
3		121.44	8.36	14.00	0.33	27.56	10.06	2.83	1.74	71.37
4		206.88	5.19	206.87	12.31	35.04	10.31	4.27	1.77	103.08
5		193.38	14.10	27.62	11.98	21.81	7.97	3.00	1.61	85.95
6		129.50	nd	34.25	8.47	34.44	11.75	3.51	2.06	90.71
7	*Crataegi fructus*	102.00	13.36	9.31	4.11	9.54	2.94	2.76	1.31	18.45
8		41.60	6.17	11.00	7.70	9.78	2.89	1.56	1.28	28.33
9	*Helichrysi arenarii flores*	104.81	15.15	37.25	25.58	72.41	36.61	3.48	2.96	145.77
10		117.34	7.56	37.84	14.47	60.41	42.50	3.25	2.30	150.02
11		119.95	17.51	35.25	17.61	119.33	73.19	3.48	2.76	78.95
12		85.63	8.39	38.37	15.78	65.72	33.64	3.20	2.19	137.48
13		101.12	nd	33.56	7.47	93.00	31.11	3.65	1.86	72.41
14	*Sambuci nigrae flores*	224.40	13.22	35.98	19.31	28.44	9.50	3.97	2.13	84.33
15		147.92	11.31	36.38	18.53	22.35	12.08	3.77	2.44	103.06
16		10.56	0.29	49.38	20.93	32.54	15.97	5.48	2.50	107.56
17		167.69	8.38	48.94	37.01	47.00	13.53	4.76	2.75	109.22
18		120.69	10.67	44.25	20.87	24.34	16.17	5.16	2.85	93.00
19		110.94	nd	27.87	8.39	37.75	12.75	4.56	3.52	126.14
20	*Chamomille flos*	26.38	1.45	31.63	13.31	39.53	17.31	5.48	2.20	37.95
21		190.31	6.67	14.69	6.42	24.04	4.33	4.97	1.49	10.02
22		313.06	8.60	37.33	12.42	40.19	13.39	5.64	2.12	73.27
23		224.68	17.08	30.56	12.78	53.88	17.67	4.14	2.25	50.09
24		228.00	6.42	41.40	12.50	48.04	15.56	3.36	2.19	114.34
25		16.17	0.31	40.50	13.17	47.58	10.25	5.09	2.08	73.74
26		409.72	19.31	88.04	21.25	67.21	11.64	5.48	2.66	133.84
27		221.00	14.12	60.38	16.58	37.94	15.39	3.94	2.52	127.28
28		209.44	nd	29.31	6.89	45.94	11.83	3.13	1.84	46.45
29		121.56	nd	30.31	5.14	92.31	27.36	2.26	1.27	79.31
30	*Tiliae flores*	292.96	12.19	60.68	13.11	57.29	6.56	4.33	1.57	121.25
31		167.19	1.78	10.50	1.80	63.58	15.34	5.86	3.36	175.57
32		177.31	3.56	10.13	1.72	55.71	16.26	5.48	3.68	54.01
33		125.06	1.69	9.81	5.00	27.44	6.65	5.67	3.04	118.90
34		113.81	2.89	11.38	4.67	175.21	26.85	6.05	3.15	98.15
35		91.50	4.50	10.80	6.25	101.16	16.81	6.25	3.41	74.55
36		89.75	6.58	12.00	5.67	120.21	24.19	6.83	5.07	78.62
37	*Violae herba*	336.13	2.98	35.19	21.56	87.00	26.17	3.75	1.34	20.33
38		233.54	4.33	39.19	22.28	85.15	45.86	3.17	1.78	27.60
39		245.50	3.81	37.53	5.45	52.71	29.72	3.56	1.37	29.07
40		199.18	3.89	38.13	26.47	86.37	42.94	3.65	1.65	22.13
41		378.75	nd	37.81	13.19	182.56	57.47	2.05	1.49	46.61
42	*Hyperici herba*	92.17	nd	50.33	10.55	201.06	57.60	4.07	1.87	123.07
43		161.83	1.23	54.89	11.40	57.06	14.08	4.77	1.89	108.51
44		50.22	nd	44.00	8.35	67.05	23.3	4.08	1.84	107.23
45		94.83	nd	51.22	10.18	148.56	44.38	4.38	1.78	191.78
46		44.39	nd	45.22	10.13	74.17	29.00	4.07	2.05	125.64
47		55.00	nd	49.20	8.20	66.5	22.35	5.04	1.97	185.13
48		45.39	nd	43.00	13.53	187.11	94.93	3.32	1.57	100.48
49		129.22	nd	42.90	12.60	75.39	34.88	3.82	2.07	111.25
50		65.38	nd	45.56	9.20	101.17	33.45	4.11	2.03	148.75
51		77.06	nd	30.94	11.61	103.56	38.17	2.91	2.23	133.54
52	*Polygoni aviculare herba*	418.13	1.94	20.81	3.44	50.17	10.76	5.48	2.72	30.46
53		237.12	1.14	11.69	6.53	33.52	12.46	5.19	2.61	28.09
54		356.88	1.25	16.25	8.28	37.10	14.33	5.86	2.83	34.62
55		509.38	3.25	16.50	4.39	85.84	24.59	5.96	2.51	24.15
56		405.62	4.19	10.31	4.06	43.81	19.07	6.05	2.83	35.44
57		283.88	nd	28.94	8.75	54.38	17.94	2.04	1.44	43.02
58	*Equiseti herba*	263.38	nd	39.68	2.00	54.27	18.47	6.25	2.40	74.84
59		68.88	1.08	37.50	1.78	21.71	17.91	5.57	2.63	52.86
60		91.00	1.86	36.81	2.10	57.73	29.42	6.15	2.83	45.78
61		166.94	nd	25.25	1.64	37.12	15.69	5.28	2.56	54.90
62		121.31	1.17	20.69	1.50	36.23	18.57	5.67	2.51	53.18
63		355.04	0.22	19.94	1.25	33.35	14.86	5.60	2.64	46.55
64		128.62	0.23	19.25	0.97	42.88	21.89	5.57	2.71	32.39
65		71.43	0.75	24.00	5.72	54.72	28.04	6.44	3.26	36.16

Arithmetic mean of three measurements is presented

*ex* water-extractable forms of metallic elements, *nd* not detected (below LOD)

The results of determinations of metallic elements essential for human health are consistent with the data found in literature [6–9]. The fact that several botanical plant species contained similar level of Fe, Zn, Cu, and Mn was revealed also by literature data. For example, other researchers after studying the elemental content in four species of *S. officinalis* has found that level of Cu, Mn, Zn, and Fe differs significantly in them [14]. Level of metals in soil can also have an impact of the concentration of these elements in medicinal plants; however, the massive effect of botanical plant species on the level of metals was noticed, too [10, 11].

To verify whether the differences between the mean concentrations of Fe, Mn, Zn, and Cu as well as in the total flavonoids contents are statistically significant, one-way ANOVA was applied. ANOVA revealed that only in the case of two metallic elements, namely total Cu and water-extractable Fe, the differences between the samples originating from Belarus and those from Ukraine and Romania are significant (α < 0.05). This was confirmed by post hoc Tukey test, where the calculated values of *p* were much lower than confidence level of 0.05. As for the differences between the level of other remaining metallic elements and total flavonoid contents, they were insignificant.

In the next step, it was investigated whether the differences among the studied analytes, owing to the fact that analyzed herbal drugs belonged to various botanical plant species, were significant. It was found that the number of statistically significant differences is much higher when compared with the differences due to the origin from three countries of Eastern Europe. For example, the level of Fe differs significantly in herbs from *Polygoni aviculare herba* and all other botanical plant species. In fact, only in the case of Zn level the differences among various botanical plant species were not statistically significant (α < 0.05). As noticed for other metals and their water-extractable forms, these differences were significant, similarly as for total flavonoid contents.

Correlation analysis has revealed several significant relations among pairs of metals, as shown in Table 3. The most frequent correlations occurred between water-extractable forms of Fe, and total Mn, water-extractable Mn, also total Cu, water-extractable Cu, and between water-extractable Fe and total flavonoids. On the other hand, total flavonoids contents was significantly related to total and water-extractable Fe, also to total and water-extractable Zn, as well as to total Mn. The correlations between total and water-extractable forms were found for three metals, Zn, Mn, and Cu. Especially high statistically significant correlation (*r* = 0.80; α < 0.05) was revealed for the pair: total Mn − water-extractable Mn. Analyzing the correlations between the pairs of metals, the statistically significant relations between total and water-extractable forms of metals were revealed before in other studies [7, 8]. Correlations between concentrations of different metals can be explained by their participation in the same biochemical pathways in plant organism [12]. However, the negative correlation obtained in this study, e.g., between the level of water-extractable Fe and water-extractable Cu and total Cu, can be explained by the fact that both metals are electro-active, and perhaps they participate in the same interactions with flavonoids, as described in a recent research [19]. The same type of interactions among certain flavonoids and metallic elements could occur in the studied herbal drugs, since they are rich in different flavonoid compounds.

**Table 3:** Correlation matrix

	Fe total	Fe ex	Zn total	Zn ex	Mn total	Mn ex	Cu total	Cu ex	Total flavonoids
Fe total	1								
Fe ex	−0.16	1							
Zn total	0.01	0.09	1						
Zn ex	−0.01	−0.04	*0.36*	1					
Mn total	−0.05	*0.45*	0.03	0.09	1				
Mn ex	−0.15	*0.41*	0.06	0.22	*0.80*	1			
Cu total	0.13	*−0.36*	−0.12	−0.29	−0.06	−0.21	1		
Cu ex	−0.03	*−0.32*	−0.25	−0.15	−0.01	−0.09	*0.72*	1	
Total flavonoids	*−0.35*	*0.29*	*0.33*	*0.25*	*0.25*	0.18	−0.05	0.13	1

Statistically significant correlation coefficients (α < 0.05) are in italics

*ex* water-extractable species

**Fig. 2:**
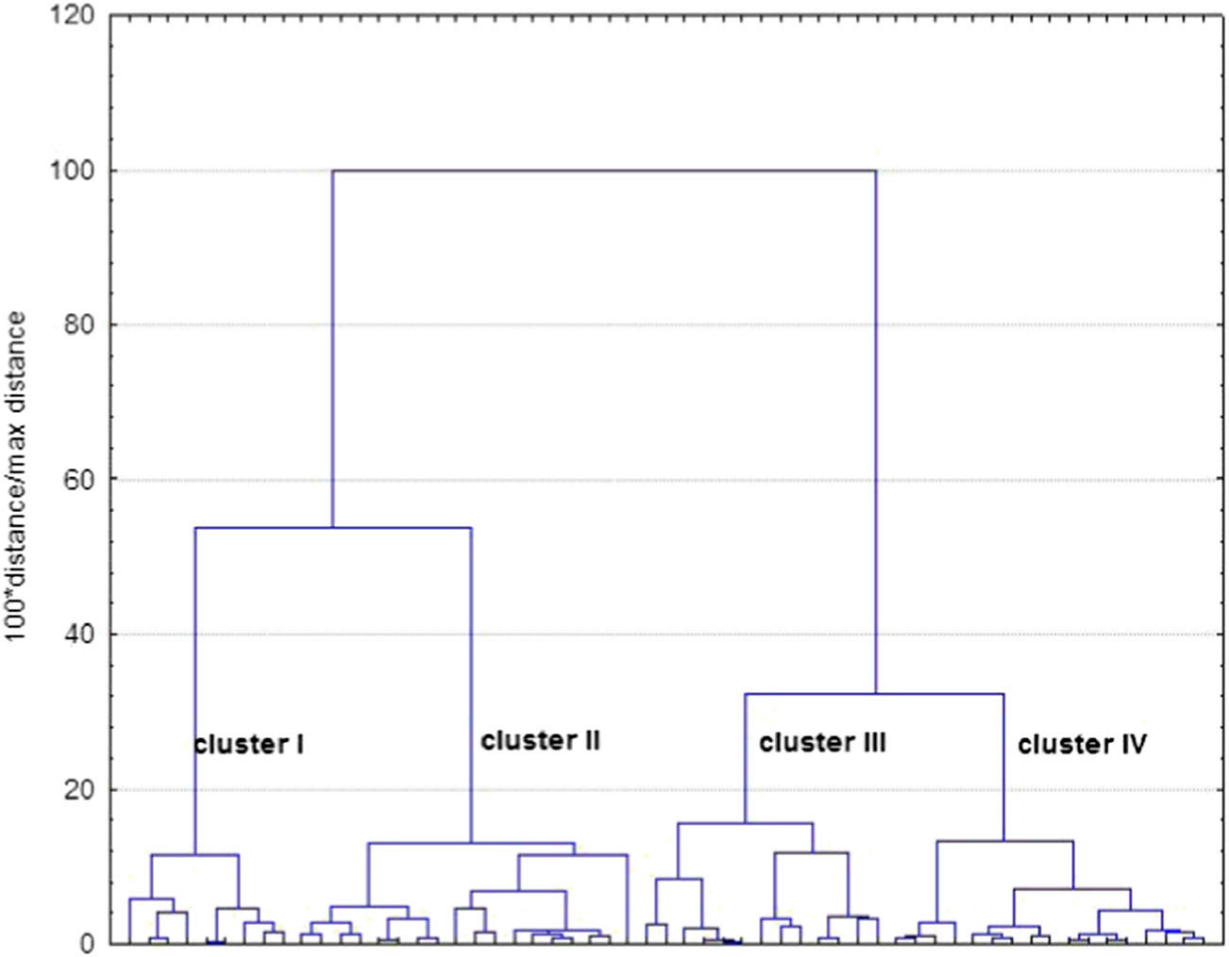
Dendrogram showing cluster analysis results for analyzed medicinal herbs

The CA has grouped the studied herbal drugs into several clusters, including herbal samples with similar level of analyzed elements and total flavonoids. Figure 2 shows four well-separated clusters, numbered I–IV, which contain characteristic herbal samples. For example, in cluster I, there are most of *Hyperici herba*, with the numbers of 45, 47, 48, and 49, and single samples of herbs from other botanical species. On the other hand, in the far right cluster IV, it is possible to notice the samples of 1, 2, and 3 which are *Crataegi folium et flores*, as well as samples 7 and 8, which are *Crataegi fructus*. Such examples of grouping together the samples with similar chemical composition (level of metals and total flavonoids) can be numerous, and quite often, it is possible to see that these samples belong to the same botanical plant species. Thus, our CA results are comparable to those obtained in a study of trace metal concentrations in some herbs and herbal teas, where CA has grouped the herbal samples based on their levels of elements [20]. It was also found, similarly as in our study that seven groups of herbs contain samples which belong to different botanical plant species, as notice in the case of nettle and senna, chamomile, peppermint, lemon balm, sage, and other species.

**Fig. 3:**
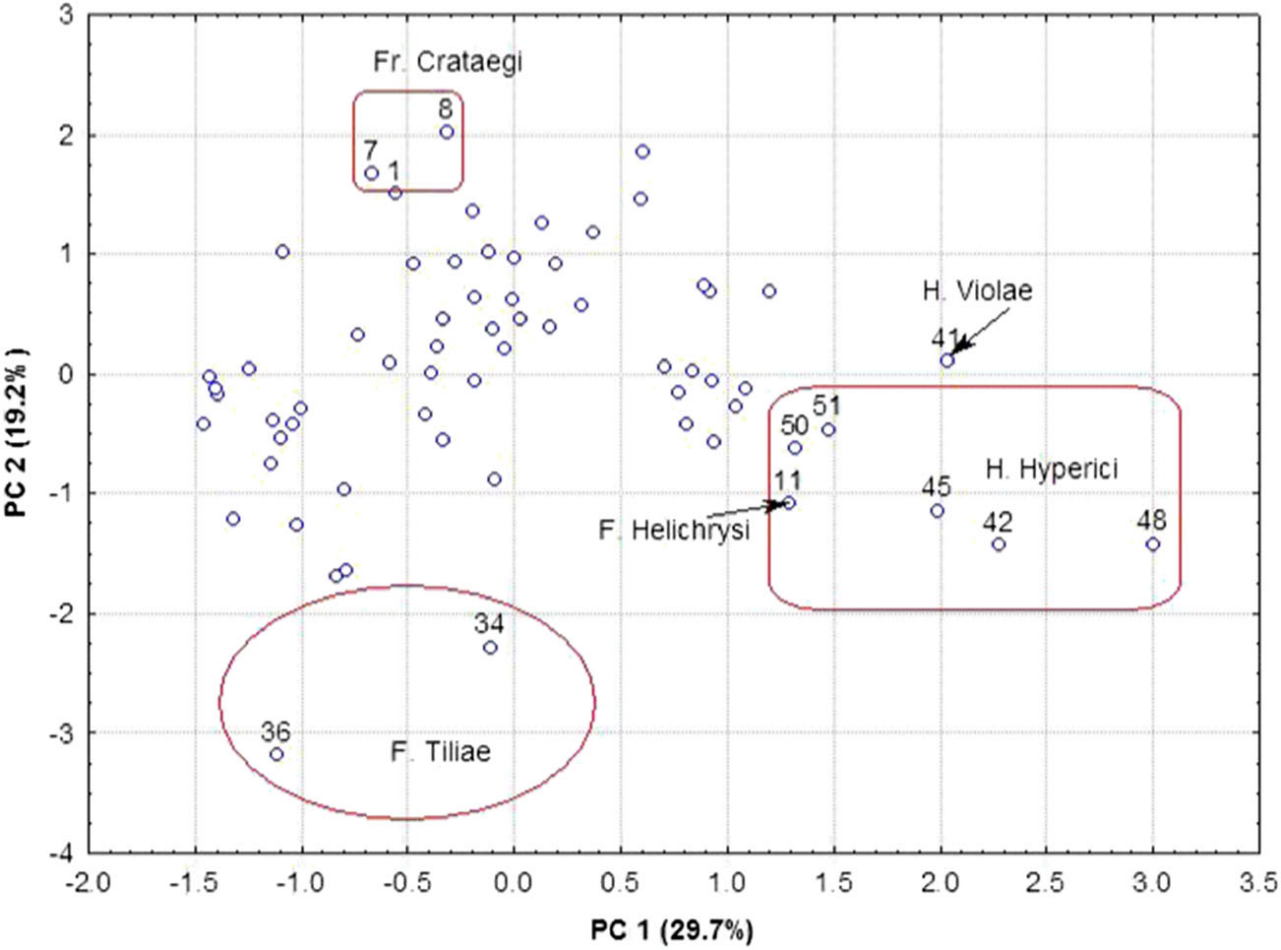
Scatterplot of herbal samples in two-dimensional areas PC1 and PC2

The PCA was applied to reduce multidimensionality in the examined database and to find the factors responsible for differentiation of the studied herbal samples. PCA calculation showed that two first principal components (PC1 and PC2) are responsible for almost 49% of variability among the studied herbal samples. Figure 3 shows the scatterplot of the samples in two-dimensional areas PC1 and PC2, and some characteristic groups were identified. In the right area of the plot, there is a group of *H. herba*, which can be easily related to cluster I in CA. On the other hand, in the higher area of the plot, there are several samples of *Crataegi fructus*, and in the lower part of the plot, one can find some samples of *Tiliae flos*.

**Fig. 4:**
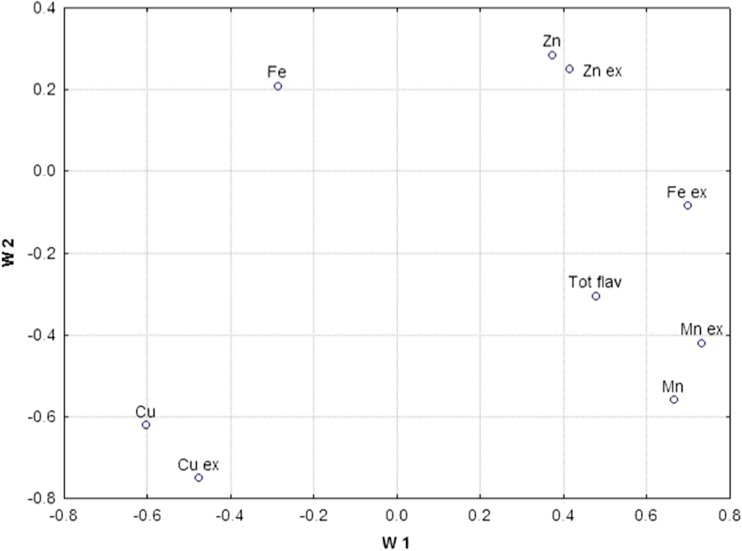
Loading plot for studied herbal samples in two-dimensional plots W1 and W2

Figure 4 illustrates the loadings of principal components. It follows that PC1 is highly correlated with the level of total and water-extractable Mn, and with water-extractable form of Fe, whereas PC2 is correlated to total and water-extractable Cu.

Geographical origin of black tea samples was investigated to demonstrate its impact on the level of macro- and microelements, and it was found, similarly as in our studies, that differentiation of teas was possible based on concentrations of metallic elements [21]. Moreover, statistical methods, such as cluster analysis and factor analysis, appeared to be good tools, which enabled to differentiate samples in terms of their provenance, as in our investigation. Another study, where determination of geographical origin of Chinese medicinal plant, *Marsdenia tenacissima*, was important subject of investigation, confirms the fact that chemometric tools, such as ANOVA and PCA are necessary to find the proper relations between the level of elements and cultivation area in certain provinces or regions [22]. An integrated approach for determining the geographical origin of medicinal herbs was used by the researchers from South Korea [23]. They have also found that instrumental techniques, such as ICP-AES/ICP-MS and as well as ^1^H NMR in combination with four classification techniques (LDA, KNN, SVM and PLS-DA), are helpful in identification of sources of differentiation of medicinal plants. However, it must be emphasized that in our study, a more important factor causing the differentiation of medicinal herbs based on elemental contents, was their botanical species, not the geographical origin of medicinal plants. The exception was the difference in level of water-extractable Fe and total Cu, significantly different in samples originating from Ukraine and Romania. This could support the findings of other researchers [20–23] that the impact of geographical origin is crucial for differentiation of medicinal plant samples.

## Conclusions

First of all, performed analyses of metallic elements essential for human life (Fe, Zn, Mn, and Cu), as well of total flavonoid contents, allow us to state that these plants are rich sources of studied metals and flavonoids. Moreover, several medicinal herbs contained significantly high amounts of metals, for example *Hyperici herba*, *Tiliae flores*, and *Crataegi fructus*. On the other hand, ought to the application of multivariate statistical methods, such as CA and PCA, it was possible to classify the studied herbs into clusters with characteristic botanical plant species and with similar level of analytes. Analysis of variance has revealed the massive impact of botanical plant species on the level of metals and total flavonoids. In the case of herbal drugs originating from Belarus, it was found by ANOVA tests that the level of water-extractable Fe and total Cu was significantly different in them in comparison with medicinal plants from Ukraine and Romania. In this way, the impact of geographical origin of analyzed plants on the level of studied elements was revealed.
